# Correction to: Risk perception related to COVID-19 among the Iranian general population: an application of the extended parallel process model

**DOI:** 10.1186/s12889-021-11903-5

**Published:** 2021-11-03

**Authors:** Leila Jahangiry, Fatemeh Bakhtari, Zahara Sohrabi, Parvin Reihani, Sirous Samei, Koen Ponnet, Ali Montazeri

**Affiliations:** 1 Tabriz Health Services Management Research Center, Tabriz University of Medical Sciences, Tabriz, Iran; 2 Health Education and Health Promotion Department, School of Public Health, Tabriz University of Medical Sciences, Tabriz, Iran; 3 Medical Education Research Center, Health Management and Safety Promotion Research Institute, Tabriz University of Medical Sciences, Tabriz, Iran; 4 Faculty of Social Sciences, Imec-mict Ghent University, Ghent, Belgium; 5 Population Health Research Group, Health Metrics Research Center, Institute for Health Sciences Research, ACECR, Tehran, Iran; 6 Faculty of Humanity Sciences, University of Science and Culture, Tehran, Iran

**Correction to: BMC Public Health (2020) 20:1571.**

http://orcid.org/10.1186/s12889-020-09681-7

It was highlighted that in the original article [1], in the Methods and Results sections of the Abstract, the word “threat” was erroneously written as “treat” in the below two sentences. The original article has been updated.

## Abstract (methods)

To collect data an electronic self-designed questionnaire based on the EPPM was used in order to measure the risk perception (efficacy, defensive responses, perceived threat) related to the COVID-19.

## Abstract (results)

The results revealed significant differences in efficacy, defensive responses, and perceived threat among different population groups particularly among those aged 60 and over.
